# BIOPHYTIS BIO101 IN SARCOPENIA: UPDATE ON THE SARA PROGRAM: FROM SARA-INT TOWARDS THE PHASE 3 STUDY

**DOI:** 10.1093/geroni/igac059.2934

**Published:** 2022-12-20

**Authors:** Cendrine Tourette, Waly Dioh, Carole Margalef, Jean Mariani, Sam Agus, Rob Van Maanen, Stanislas Veillet

**Affiliations:** Biophytis SA, Paris, Ile-de-France, France; Biophytis, Paris, Ile-de-France, France; Biophytis, Paris, Ile-de-France, France; Biophytis, Paris, Ile-de-France, France; Biophytis Inc., Cambridge, Massachusetts, United States; Biophytis, Paris, Ile-de-France, France; Biophytis, Paris, Ile-de-France, France

## Abstract

Sarcopenia is a progressive muscle disorder increasing with age that may lead to mobility disability. SARA program strives to develop a viable option to treat community dwelling older adults suffering from sarcopenia. SARA-INT is a randomized three-arm interventional study (BIO101 175 mg bid / BIO101 350 mg bid / placebo) with treatment duration of 6 months. Eligibility criteria for sarcopenia were meeting FNIH criteria and Short Physical Performance Battery (SPPB) score ≤ 8/12 in men and women aged ≥ 65 years; primary endpoint was the 400-meter walking test (400MWT). 233 participants aged 65 years and older were randomized, 232 and 156 participants were included in the Full Analysis Set (FAS) and Per-Protocol (PP) populations, respectively. Due to COVID-19 pandemic, most end-of-treatment efficacy assessments are missing for 55% of the participants, reducing the studies’ power. BIO101 350 mg bid treatment led to an improvement in the primary endpoint, the gait speed from the 400MWT of 0.07 m/s in the FAS population (not statistically significant) and of 0.09 m/s in the PP population (nominally statistically significant, p=0.008) after 6 months; this is close to MCID in sarcopenia (0.1 m/s). BIO101 350mg bid treatment effect on the 400MWT is confirmed in PP sub-populations at high risk of mobility disability. Trends were observed with other endpoints. BIO101 showed a very good safety profile at both doses. Biophytis will initiate the phase 3 program by end 2022, targeting a severe sarcopenic population. Outcomes of the interactions with regulatory agencies on study design will be presented.

